# Preferential enhancement of laser-driven carbon ion acceleration from optimized nanostructured surfaces

**DOI:** 10.1038/srep11930

**Published:** 2015-07-08

**Authors:** Malay Dalui, W.-M. Wang, T. Madhu Trivikram, Subhrangsu Sarkar, Sheroy Tata, J. Jha, P. Ayyub, Z. M. Sheng, M. Krishnamurthy

**Affiliations:** 1 Tata Institute of Fundamental Research, Homi Bhabha Road, Colaba, Mumbai 400 005, India; 2 Beijing National Laboratory for Condensed Matter Physics, Institute of Physics, Chinese Academy of Sciences, Beijing 100190, China; 3 Forschungszentrum Jülich GmbH, Institute for Advanced Simulation, Jülich Supercomputing Centre, D-52425 Jülich, Germany; 4 IFSA Collaborative Innovation Center, Shanghai Jiao Tong University, Shanghai 200240, China; 5 SUPA, Department of Physics, University of Strathclyde, Glasgow G4 0NG, United Kingdom; 6 Key Laboratory for Laser Plasmas (MoE), Department of Physics and Astronomy, Shanghai Jiao Tong University, Shanghai 200240, China; 7 TIFR Centre for Interdisciplinary Sciences, 21 Brundavan Colony, Narsingi, Hyderabad 500075, India

## Abstract

High-intensity ultrashort laser pulses focused on metal targets readily generate hot dense plasmas which accelerate ions efficiently and can pave way to compact table-top accelerators. Laser-driven ion acceleration studies predominantly focus on protons, which experience the maximum acceleration owing to their highest charge-to-mass ratio. The possibility of tailoring such schemes for the preferential acceleration of a particular ion species is very much desired but has hardly been explored. Here, we present an experimental demonstration of how the nanostructuring of a copper target can be optimized for enhanced carbon ion acceleration over protons or Cu-ions. Specifically, a thin (≈0.25 μm) layer of 25–30 nm diameter Cu nanoparticles, sputter-deposited on a polished Cu-substrate, enhances the carbon ion energy by about 10-fold at a laser intensity of 1.2×10^18^  W/cm^2^. However, particles smaller than 20 nm have an adverse effect on the ion acceleration. Particle-in-cell simulations provide definite pointers regarding the size of nanoparticles necessary for maximizing the ion acceleration. The inherent contrast of the laser pulse is found to play an important role in the species selective ion acceleration.

The interaction of high intensity (≥10^18^ W/cm^2^) lasers with solids and the subsequent ion acceleration is a rapidly growing research field with possible applications in fundamental and technological areas such as medical physics^1^, high energy density science^2^, laboratory astrophysics^3^, ion-driven fast ignition inertial confinement fusion^4^,^5^, relativistic ion beam production^6^ and proton deflectometry^7^.

An intense, ultrashort laser pulse, incident on a solid surface produces hot electrons. During or immediately after the interaction of the laser pulse with the surface, the motion of ions is negligible compared to that of electrons, as the former are much more massive. The resulting charge separation leads to the formation of a quasi-static sheath electric field. This electric field accelerates the ions at slightly longer time scales (~100 fs), depending on their charge-to-mass ratio (q/m)^8^,^9^,^10^ and results in a compact as well as effective ion acceleration. The higher the laser intensity, the more efficient is the hot electron generation, which has a direct influence on the ion acceleration. However, a large portion of the incident light is reflected from the critical density layer of the plasma (where the laser frequency becomes equal to the plasma frequency and the laser cannot propagate deeper into the plasma) and only a small percentage of the laser energy is transferred to the electrons and even lesser to the ions^11^. In typical laser-solid experiments, the vacuum level of the experimental chamber (typically 10^−5^ Torr) is not too high, such that the water vapour and/or hydrocarbons adsorbed on the target surface invariably generate ion species such as H^+^, C^*q*+^, O^*q*+^ (*q*= charge state) in addition to the metal ions from the bulk target^12^,^13^. Protons, being the lightest of the ions, are easily detected and are almost exclusively studied to probe the acceleration physics. The laser energy transfer to the heavier ions needs to be increased significantly if viable applications are to be feasible. One of the simplest ways is to use structured targets with surface modulations of the order of or less than the laser wavelength. Although, the use of such targets to boost the ion acceleration was predicted earlier using particle-in-cell simulations^14^,^15^, more recently Margarone *et al.* have demonstrated a 66% increment in the highest proton cut-off energy and 5 times higher yield of protons with energies more than 1 MeV using nanostructured targets^16^. From a study of targets based on monolayers of polystyrene spheres of diameters 266, 535 and 920 nm coated on plastic substrates, the acceleration was reported to be optimal for 535 nm spheres. On the other hand, ellipsoidal microparticle-coated targets have yielded a 10-fold enhanced ion emission^17^. However, the effect of structuring the target surface (at the level of ∼100 nm or less) on the ion acceleration has hardly ever been explored.

Targets coated with nanoparticles or microparticles are known to absorb much more laser energy than polished flat surfaces^18^,^19^. Experiments have demonstrated either an increase in the hot electron yield or an enhanced x-ray emission, but how the acceleration of the ionic species is affected by such modifications on the target surface has, however, been explored relatively less. Bagchi *et al.* have demonstrated that in spite of having higher x-ray yield, the maximum ion energy from the nanoparticle-coated targets is less than from polished substrates^20^,^21^. The average size of the nanoparticles in these experiments was 15 nm and a time-of-flight (TOF) technique was employed to measure the ion energy spectra. The lack of charge or mass resolution is a serious impediment in the attempt to address the multi-species ion acceleration from nanostructured targets. The TOF signal, is actually a convolution of the energy spectra of every individual ionic species. Hence, the TOF provides very little insight on the species which are being accelerated and, in turn, on the underlying physics of the acceleration scheme.

The question we address in this report is whether the ion acceleration with nanoparticle-coated metals is similar for all the ionic species; if not, are any specific ions preferentially accelerated to a greater degree than protons and metal ions? Our present experiments clearly show that the ion acceleration is indeed dependent on the ion species (q/m) and an appropriately designed nanostructured coating is much more efficient in accelerating carbon or oxygen ions as compared to protons or Cu ions. We find that if the average crystallite size is smaller than 20 nm, the accelerations of all the ionic species are much lower than from a polished copper target. On the contrary, when the polished Cu-substrate is coated with a moderately thick (≈0.25 μm) layer of Cu-nanoparticles (of 25–30 nm average size), the acceleration of carbon ions, in particular, increases dramatically to values as large as an MeV. This is in stark contrast to plain (unstructured) solid targets, which yield at best 90 keV carbon ions. However, the maximum detected energies of protons or Cu ions are similar or only marginally higher for targets coated with 25–30 nm sized nanoparticles.

A quantitative understanding of the effect of nanostructuring on the ion acceleration can be obtained from a fully relativistic, two dimensional particle-in-cell (PIC) simulations. The results indicate that for particles smaller than 20 nm, the ion acceleration is less efficient and a larger particle size is necessary to boost the acceleration. To explain the preferential acceleration of carbon ions, we have considered the ion motion from the target surface under the influence of the laser pre-pulse. MULTI-fs hydrodynamic simulations show that protons, being less massive than C or Cu ions, move ahead of the C ions before the arrival of the main pulse. We conjecture that protons are correspondingly less accelerated in the electrostatic sheath field compared to the carbon ions as their accelerating length is reduced. The Cu ions are much more massive than the C ions and their relative motion is much less. However, as the lighter ions move, the sheath field gets lowered and the acceleration of the much heavier Cu ions is hindered. Our analysis based on the long range temporal profile of the laser brings out the enhanced carbon ion acceleration demonstrated in this report. Our experimental observations clearly indicate that the acceleration is not uniform for all the ionic species and the acceleration dynamics can be optimized for a particular species for a given set of laser parameters. The results presented in this report, therefore, should be of great interest for making viable, laser-plasma based ion accelerators.

## Results

**Figure 1:**
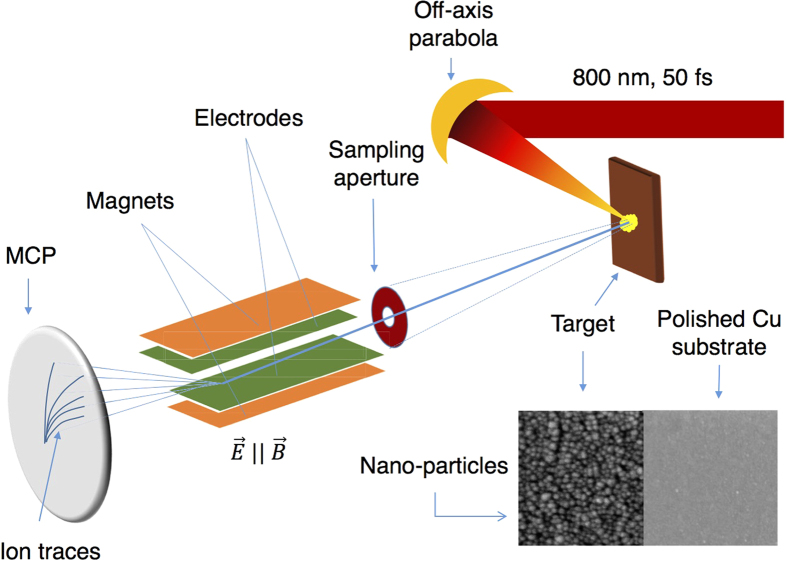
Schematics of the experimental set up. A 800 nm, 50 fs, *p*-polarized laser beam is focused on the target at an intensity of 1.2 × 18 W/cm^2^. The target is a Cu-substrate and on half of it Cu-nanoparticles are sputter-deposited as shown in the inset. The ion beam, sampled through an aperture is diagnosed by a Thomson parabola spectrometer (TPS). The parabolic ion traces on the micro-channel plate (MCP) is imaged by a CCD camera.

**Figure 2:**
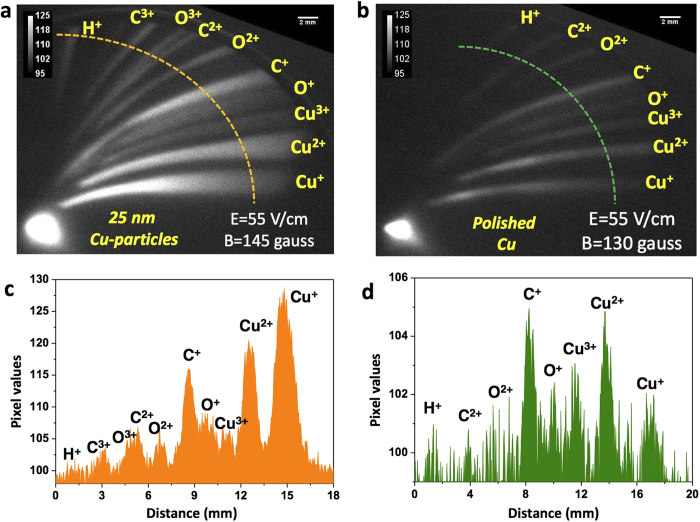
Thomson parabola ion traces. TPS images (**a**,**b**) from the 25 nm Cu-particle coated target and the reference Cu-substrate respectively at 1.2 × 10^18^ W/cm^2^ laser intensity. The horizontal axis gives the deflection due to the electric field and the vertical axis is due to the magnetic field of the TPS. The applied electric and the magnetic field values are shown in the corresponding ion trace images. **c**,**d** shows a line profile through the TPS ion traces for both the targets. Each ionic species are labelled correspondingly.

**Figure 3:**
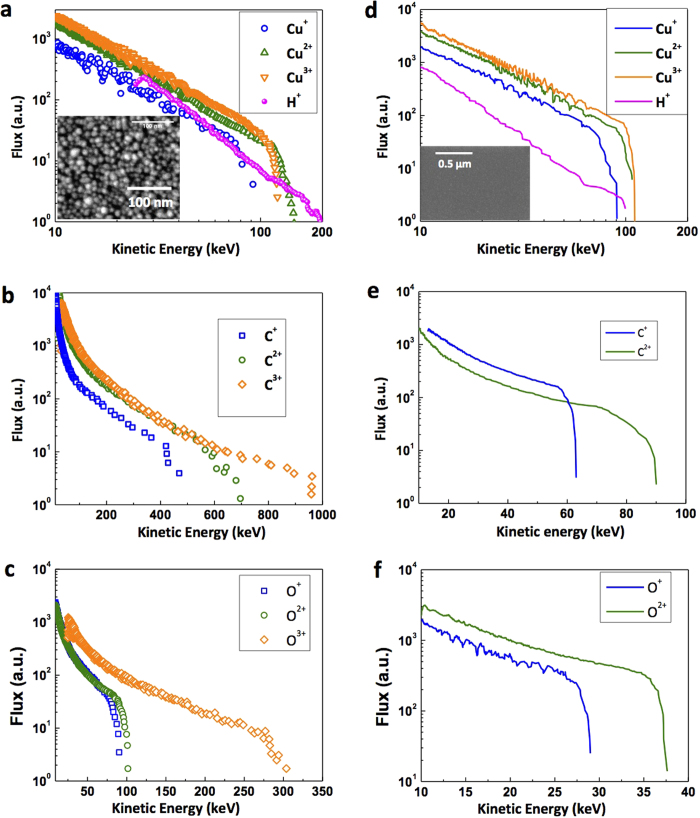
Energy spectra. Ion energy spectra for all the different ions obtained from the TPS images of both the nanoparticle (of 25 nm average size) coated target (left panel: **a**–**c**) and the polished Cu-substrate (right panel: **d**–**f**). Inset in **a** and **d** shows the corresponding SEM images of the targets. A 10 fold increment in the maximum carbon ion energy and an 8 fold increment in the maximum oxygen ion energy is observed with the nanoparticle coating compared to the polished bulk surface.

Ion emission measurements were carried out from the front surface of polished Cu-targets and compared each time with those from Cu-nanoparticle coated targets to understand the systematic variation in ion generation for mean particle sizes in the 7–25 nm range, as illustrated in figure 1. The ion emission from the target coated with a nanocrystalline Cu film with a mean particle size of 25 nm shows a rather drastic change compared to that from the polished bulk Cu-substrate. The laser energy coupling in this sample is enhanced significantly with respect to the polished Cu-substrate. Figure 2 shows the Thomson parabola ion traces from the nanoparticulate target and the polished Cu target. Cu-ions up to Cu^3+^ were observed from both the targets. A line cut of the image along the dotted line shows the well resolved ion peaks. Figure 3 shows the ion energy spectra obtained by analyzing Cu^*q*+^, H^+^, C^*q*+^ and O^*q*+^ ion traces of the TPS image^22^. For all the ions, the maximum energy is found to be significantly larger from the nanoparticulate target than from the reference. For instance, the maximum proton energy in the reference is ≈100 keV, whereas it is almost 200 keV from the 25 nm nanoparticle coated target, implying a two-fold increase in the proton acceleration. The proton flux in the energy range 25–100 keV is also about 1.5 times higher in the nanoparticulate target, but the Cu-ion flux is slightly lower than that of the reference Cu-substrate. The most significant enhancement in the acceleration occurs in the case of the carbon ions. Figure 3b,e show the carbon ion energy spectra from the 25 nm nanoparticle-coated target and the polished Cu target, respectively. The highest cut-off energy for C^2+^ and C^3+^ are 700 keV and 950 keV respectively from the nanoparticulate target, whereas, the highest detected carbon ion energy from the bulk Cu-substrate is only about 90 keV. Thus, coating the bulk Cu target with a layer of nanoparticles with an average size of 25 nm is found to provide more than 10-fold enhancement in carbon ion acceleration. Oxygen ions also exhibit similar acceleration features (8 fold enhancement in the maximum ion energy compared to the bulk), as shown in figure 3c,f. The highest observed oxygen ion energy is however less than that of the carbon ions for the same targets (nanoparticulate and polished).

**Figure 4:**
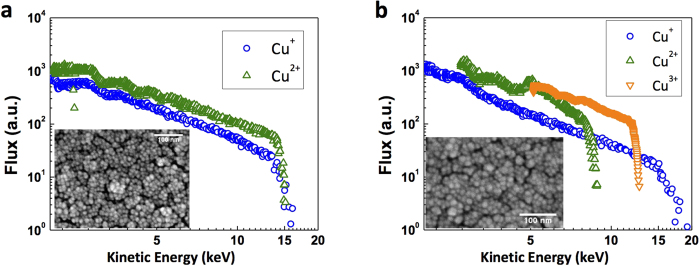
Energy spectra. The copper ion energy spectra (**a**,**b**) from 7 nm and 15 nm particle coated targets respectively measured using the TPS images. Inset shows the SEM images of the corresponding targets.

**Figure 5:**
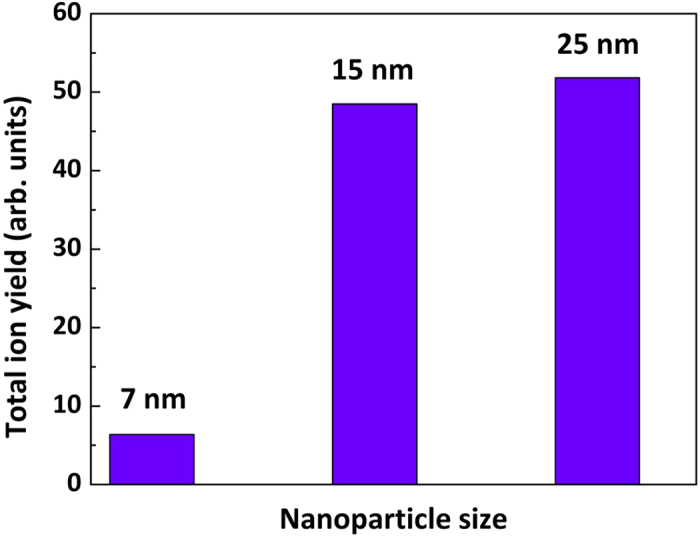
Ion yield. Total ion yield from the nanoparticle (of different sizes) coated targets. The size of the particles are labelled accordingly.

Interestingly, the nanoparticle-coated targets with relatively smaller particles (average size ≈ 7 nm and 15 nm) yielded negligible ion acceleration compared to the polished Cu-target. Figure 4 shows the kinetic energy spectra of the Cu-ions from the 7 nm and 15 nm sized nanoparticle coated targets. Irradiation on the 7 nm particle coated target yields up to doubly charged states of Cu-ions with energies in the range 3–20 keV. Although, Cu^3+^ ions are detected from the 15 nm particles coated target, the ion acceleration does not improve appreciably. No protons or the carbon ions are detected in the TPS from either the 7 nm or 15 nm particles. The TPS has a low energy cut-off determined by the applied electric field and deflects lower energy ions out of the detector. The absence of protons or carbon ions indicate that their energies are lower than the TPS cut-off energy, which is ≈3 keV in this particular configuration. Very low energetic ion emission from smaller nanoparticle (SNP: <20 nm) coated targets could be either because of longer plasma expansion into the vacuum or ablation of such tiny structures by the pre-pulse or both. SNPs are found to absorb the low intensity light much more efficiently than the larger sized nanoparticles (LNP: >20 nm). Since the melting temperature decreases with decreasing size^23^, the pre-pulse which is about 10^−6^ times intense compared to the main pulse, significantly affects SNP (than LNP) and generates a larger pre-plasma which affects the ion acceleration. Figure 5 shows the variation of the total ion yield with the size of the coated nanoparticles. With increasing nanoparticle size from 7 nm to 25 nm, the total ion yield increases systematically, a trend that is confirmed by the PIC simulations.

A quantitative understanding of the effect of the nanoparticle coating on the ion acceleration was obtained from two dimensional PIC simulation code KLAPS^24^,^25^. Simulation results confirm that the ion acceleration is maximum for an optimal nanoparticle size. In the simulation, a laser pulse with the same parameters as used in the experiment is taken: λ = 800 nm (wavelength), τ = 50 fs (pulse duration), *p*-polarized, 45^°^ angle of incidence and a peak intensity of 1.2×10^18^ W/cm^2^. To save the computational cost, the laser focal spot size is taken to be 6.7 μm. The target is taken as a plasma composed of electrons and C^2+^ ions of density 20n_c_ with a depth of 1.6λ, where, *n*_*c*_ ≈ 1.72 × 10^21^ cm^−3^. We have not considered the case of mixed ions as the modelling of the simultaneous ionization of Cu, C, O, H atoms is difficult. The ratio of each species as well as the distribution of their positions needs to be assumed and it can generate arbitrariness in the computational results. As the interaction of the laser with the target surface and the process of ion acceleration with one species is similar to the case with several species, to understand the enhanced carbon ion acceleration, only one ionic species, namely C^2+^, which is present both in the polished and the nanoparticulate target, is taken in the PIC simulation. We model the nanoparticles as rectangular protrusions on the solid surface. The width of the protrusion denotes the size of the nanoparticles and the height of the protrusion is set to about 50 nm. The resolution of the simulations along *y*-direction (the target surface tangential direction) is 5 nm and the one along *x*-direction is 20 nm. The simulation box is 2000 × 10240 or 50λ × 64λ (*x* and *y*-directions). Simulations are performed with a time step of *δt* *=* *δy/2c* (*c* is the speed of light in vacuum). The particle number per cell is 9 for each species. Therefore, the solutions of *δt*, *δx* and *δy* satisfy the stability condition of the Maxwell equation solver. We set the initial temperatures of electrons and ions to be 30 eV according to our hydrodynamic simulation, which takes care of the target heating by the ASE (amplified spontaneous emission). Note that since the scheme for charge conservation^26^,^27^ has been adopted while calculating the currents in our PIC code, artificial numerical heating can be ignored.

**Figure 6:**
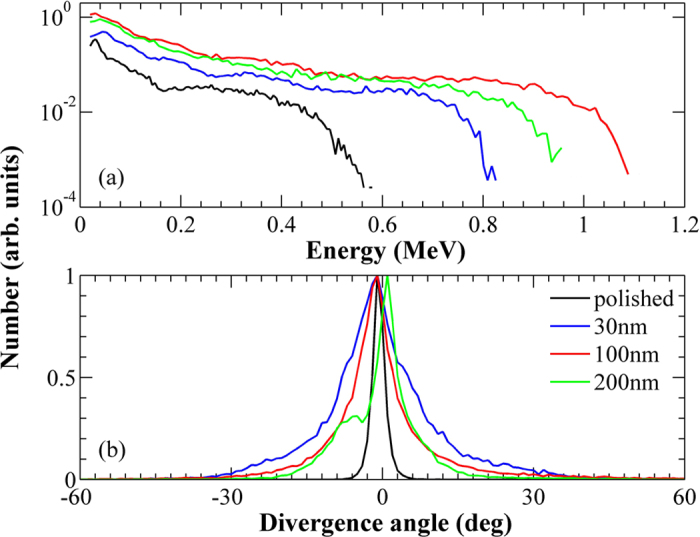
PIC simulation results. Computed ion energy spectra and ion divergence. Only the ions fleeing to the front vacuum and with energy greater than 20 keV are included in the plots.

**Figure 7:**
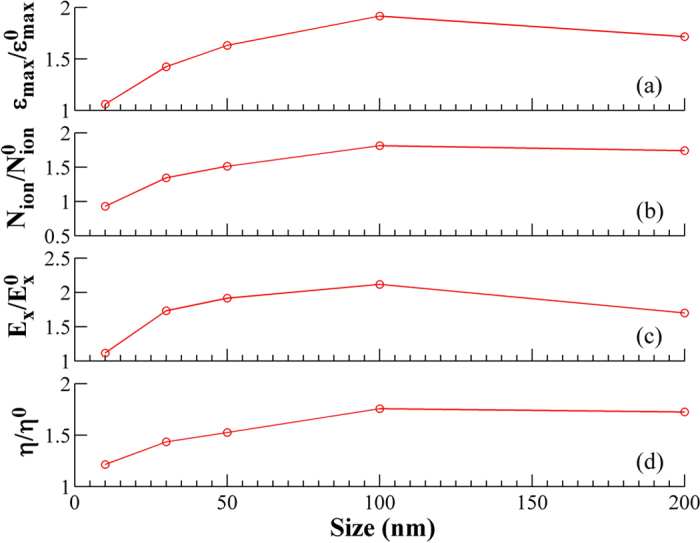
PIC simulation results. (**a**) Maximum energy of ions, (**b**) total ion number, (**c**) maximum of the accelerating electrostatic field E_X_ around the surface front and (**d**) absorption ratio of light as a function of the size of nanoparticles. All the curves are normalized with respect to the polished surface and it shows the relative size dependent enhancement with respect to that of a plain polished surface.

We found that some hot electrons start to flee the simulation box boundaries at 133 fs and therefore only the results on or before this time are valid. Figure 6 shows the result after a propagation period of 133 fs. One can see from Fig. 7 that the maximum ion energy is enhanced significantly when the nanoparticle coated surface is taken. With increasing size of the nanoparticles, the angular divergence of the ions grows considerably. The ion energy first climbs sharply from 10 nm to 30 nm, grows slowly to 100 nm and saturates or marginally decreases beyond this size. These results indicate that a nanoparticle coated surface is favorable for ion emission from the surface-front and the size of the coated particles should be optimized. Although, a peak of the maximum accelerating field appears at 100 nm, the ion number increases monotonically as the particle size is increased. It should be kept in mind that the ion divergence will also have a bearing on the ion detection normal to the surface of the target in experiments.

**Figure 8:**
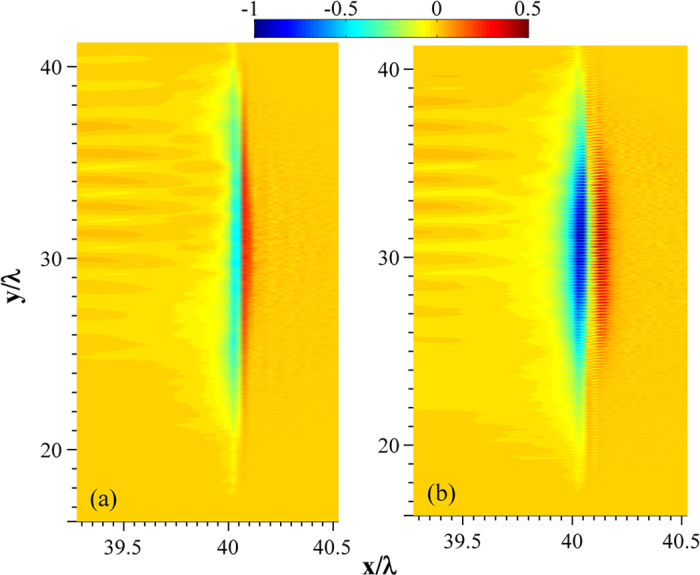
PIC simulation results. Spatial distributions of electrostatic fields, E_X_ with (**a**) a polished surface and (**b**) 100 nm-size particle coating respectively.

Generally sub-wavelength structures on the solid surface leads to enhanced laser energy absorption and higher hot electron production^27^,^28^. The spatial distribution of the electrostatic field E_X_ is shown in figure 8. Compared with the polished target, the accelerating field around the target surface front is found to be stronger and distributed over a larger area with the nanoparticles coated surface. The ion emission at the target front is mainly due to the electrostatic (ES) field E_X_. With a polished target, an ES field is formed by the charge separation mainly because the plasma electrons are pulled to the vacuum^29^. With the sub-wavelength surface structure, more electrons are pulled to the vacuum and a stronger ES field is formed^28^. Our simulation also shows that there is an optimized size of the surface structure around 100 nm, at which the ion acceleration is maximum (see figure 7). A structure of very small size, e.g., 10 nm, can be broken easily and can only provide an enhanced ES field for a short period of time, which is also observed in our simulation. Therefore, a surface structure with an appropriate size is favorable for backward emission of ions. Increasing the size beyond 100 nm, the density of the electrons pulled into the vacuum is lowered (large nanoparticle coating approaches to a polished surface), which corresponds to a reduced E_X_ around the surface front.

These computations clearly establish the trend that nanoparticles of 25–30 nm average size are better for ion acceleration compared to the smaller sized nanoparticle coated targets. Generally, producing uniform Cu-nanoparticles of 100 nm average size by the sputtering process (which is employed here) is not very easy. As a result, the experimental data are confined up to 25–30 nm, though simulations indicate that the optimum size is about 100 nm. Further investigation in this direction is in progress.

While the PIC simulation confirms the optimum target parameters responsible for maximum ion acceleration, predicting the enhancement in the carbon ion acceleration compared to protons is far more difficult. Not only is the calculation of the correct stoichiometry and spatial dependence of the C or H is a non-trivial problem, but also the computer resources required to take the effect of the motion of the ion in the pre-pulse are quite formidable.

**Figure 9:**
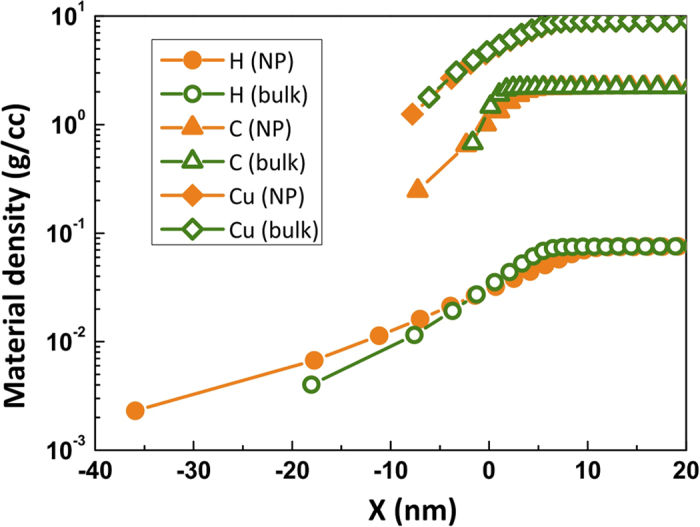
MULTI-fs hydrodynamic simulation results. The expansion of hydrogen, carbon and copper due to the pre-pulse before the arrival of the main pulse in nanoparticles coated targets and polished targets. NP is referred to as nanoparticle coated.

To understand the preferential carbon ion acceleration over the protons, we invoke the motion of the atoms from the target surface by the interaction of the laser pre-pulse. Any femtosecond laser pulse intrinsically has a temporal profile with non-zero intensity ahead of the peak of the pulse. In the present experiments, at peak intensity of 1.2 × 10^18^ W/cm^2^, the ASE level is 6 × 10^12^ W/cm^2^. It is expensive to simulate the effect of the ASE pedestal of the laser pulse, given the temporal resolution of our PIC code. At this ASE intensity, the laser energy deposition is dominated by the collisional absorption process^30^. In our PIC simulations, collisions are not included and the enhanced absorption is mainly caused by the surface structure of the target. So, we have used MULTI-fs hydrodynamic simulations^31^ to infer the details of the motion of the different ion species due to the pre-pulse. As collisions are modelled well to bridge the gap between cold metal and plasma, this code is often used to simulate the effect of the pre-pulse. In the MULTI-fs simulation, a constant pulse of 1 ns temporal duration is considered to represent the ASE. The trailing edge of the ASE is taken at 5 ps ahead of the main femtosecond pulse, where, the ASE of our laser system has an almost flat response. The target is a 25 μm thick flat substrate (of hydrogen, carbon or copper) at *x* = 0 and the laser is propagating from the left (negative *x*) to the right (positive *x*). The material density of hydrogen, carbon and copper are taken to be 0.076 g/cc, 2.2 g/cc and 8.9 g/cc respectively. Due to the lightning rod effect and the surface plasmon resonance in nanoparticles, the effective incident laser intensity on such targets increases^32^, which also increases the effective pre-pulse intensity. To make an effective comparison due to this (as compared to the plain solid slab) the nanoparticle coated target is simulated as flat targets with higher level of ASE irradiation (1.2 × 10^13^ W/cm^2^). The intensity values were taken in accordance with the PIC simulation. The results are shown in Fig. 9. Carbon and copper suffer negligible relative expansion subjected to higher ASE irradiation. However, hydrogen suffers significant expansion prior to the arrival of the main pulse. So, the ion bunch (red region in Fig. 8) gets stretched by the action of the ASE. Within the ion bunch, protons sit at the front followed by carbon, oxygen and copper ions and their spatial distributions are given in Fig. 9. The length of the charge separation field for the nanoparticulate target, as can be seen from Fig. 8(b), is ~100 nm. Hence, the length over which protons get accelerated is reduced. So, even though a larger electrostatic sheath field is generated with the nanoparticle coating, the effective proton acceleration is not that large. On the other hand, the carbon ions move very little due to the pre-pulse and experience an efficient acceleration. Cu ions being more massive also show negligible movement but the reduction of the sheath energy by the carbon and oxygen ions causes the penetration of the ES field to Cu ion positions to be less efficient, which leads to lower Cu ion acceleration.

If the pre-pulse were low enough to set the ions into motion prior to the main pulse, protons would have had the maximum acceleration followed by carbon, oxygen and Cu ions as the ES field penetration is progressively lower to the heavier ionic positions. Higher pre-pulse levels would eventually destroy the sub-wavelength structure and the nearly spherical nature of the plasma expansion may lead to inefficient ion acceleration compared to the polished bulk. Moreover, it can so happen that more than one ionic species could expand and end up at positions further for the hot electrons generated by the main pulse (dark blue region in Fig. 8b) to reach, before the ions start responding to the ES field. In that case, where the stretching of the ion bunch prior to the arrival of the main pulse is larger than the spatial extension of the ES field created by the main pulse generated hot electrons, Coulomb repulsion by the ions (at the front of the ion bunch) accelerated towards the target would reduce the ES field as well as eventually kill the acceleration of the ions situated at the back of the ion bunch, resulting negligible ion acceleration. Now, the ASE is approximately 100 times stronger at a ps (the contrast is ~10^−4^). Due to this, the spatial distribution of the ionic species would increase a bit further, but for qualitative understanding of the species preferential ion acceleration, the above analysis remains valid.

## Discussions

Although, it is now well established that structured targets enhance the laser energy coupling to the plasma and consequently increase the hot-electron as well as x-ray emission, several questions about the corresponding changes in the ion acceleration remain unanswered. Computational studies have clearly established that the proton acceleration can also be enhanced by the structured targets, be it in the form of holes in a thin foil^14^ or nanoscale modulations on the target surface^33^. However, experiments with smaller nanoparticle (~15 nm) have shown a decrease in the ion energies^20^. A well-ordered structure such as the sub-wavelength grating, on the other hand, seems to increase the maximum energy only by about 55%^34^, while nanoparticles of size close to the light wavelength or more seem to decrease the maximum proton energy^16^,^17^. On the other hand a combination of structures (from less than a μm up to 100 μm) is found to increase the proton acceleration^35^. It is therefore very important to establish the nanoparticle size dependence of the ion acceleration both experimentally and computationally. It is also clear that one cannot use the enhancement in the x-ray emission alone to predict the enhancement in the ion acceleration.

The laser energy absorption and the ion yield depend strongly on the size of the nanoparticles. They increase with the size and are optimum for a size of ≈100 nm. Beyond 200 nm, the surface starts to approximate the bulk structure and very little enhancement was observed. The laser energy deposition is also lower if the nanoparticle size is small (<10 nm). Experimentally, we have observed less ion emission for this case and the maximum ion energy is also found to be very low. Given that there is still an enhancement in the x-ray emission with the smaller nanoparticle^18^ (of 15 nm average diameter), the decrease in the ion acceleration in this case cannot be attributed to the destruction of the nanoparticles by the pre-pulse. The ES field increases by about 60% when the particle size increases from about 10 nm to 30 nm, which is the key to the enhanced ion acceleration observed by us for larger sized nanoparticles. The PIC simulations also show that even larger (≈100 nm) particles are better for the ion acceleration.

Though nanostructured targets provide better laser energy coupling and enhanced ion acceleration, the angular divergence of the accelerated ions is broader as compared to a polished surface where it forms a relatively narrow beam^15^,^18^. This effect can also be observed in the present study. Expectedly, the angular divergence of the ions is observed to be particle size dependent and becomes narrower as the size of the nanoparticles is increased. Figures 6 and 7 show all the important parameters for the ion acceleration and their variation with the nanoparticle size.

The other important questions pertain to: (a) the species dependence of the ion acceleration and (b) whether measuring the proton alone can evaluate the change in the accelerating potential due to the target surface structure. As is clear from the experimental measurements shown here, even if the proton energy increases only marginally, the energy of a heavier ion (namely carbon ion) can increase substantially. Although the effect of the ion motion during the formation of the sheath potential may be anticipated, *a priori*, it is not obvious why a ten-fold increase in carbon ion energy occurs while there is only a marginal increase in the proton energy. From a technological point of view these measurements are very important for the future application of a laser produced plasmas as an ion accelerator. Clearly, it is vital to establish the parameters which may make it possible to selectively enhance different ion species for practical applications. In this context, boosting the carbon ion energy from about 90 keV to 900 keV for the same laser parameters is significant.

## Methods

The experiment was performed using the 20 TW Ti:sapphire laser facility at the Tata Institute of Fundamental Research, Mumbai, India. The 800 nm, 50 fs, *p*-polarized laser, focused on the solid target using an off-axis parabolic mirror to a 12 μm area (FWHM) at 45^0^ angle of incidence yields a peak intensity of 1.2 × 10^18^ W/cm^2^. A thick layer of roughly spherical copper nanoparticles with controlled size was sputtered on half of a polished Cu-substrate (5 cm × 5 cm × 5 mm) which was subsequently used as the target. The nanoparticle coating was about 250 nm thick, which is large compared to the optical skin depth of the laser (~ few nm). The nanocrystalline films were grown by dc magnetron sputter deposition^35^ in argon atmosphere from a 50 mm diameter and 3 mm thick oxygen free high conductivity Cu-target. Half of all the Cu-substrate were masked and hence left unstructured, to be later used as the reference polished target. It is well known that dc/rf sputter deposition in relatively high ambient pressures and low (300 K or lower) substrate temperatures yields a nanocrystalline thin film^36^,^37^. The mean crystallite size in this case was controlled mainly by the sputtering gas (Ar) pressure. We obtained nanocrystalline Cu films with mean sizes of 25(±6) nm, 15(±2) nm and 7(±2) nm at Ar pressures of 5 mTorr, 100 mTorr and 200 mTorr, respectively. In all cases, the substrate was maintained at 300 K and the target-to-substrate separation was kept fixed at 50 mm. Deposition time was optimized for each sample such that the film thickness remained around 250 nm. The surface nanostructure was imaged using a field-emission scanning electron microscope, after plasma cleaning the surface. A piece of silicon wafer, kept alongside the substrate during deposition, was used to determine the thickness of the deposited film. The mean particle size of the deposited nanocrystalline Cu films was obtained from a statistical analysis of several SEM images. Figure 1 shows the schematics of the experimental set up. Ions, preferentially emitted normal to the target surface were measured using a Thomson parabola spectrometer (TPS) equipped with a micro-channel-plate (MCP) as the position sensitive detector and the necessary imaging optics^38^. A CCD camera, looking at the phosphor screen, was used to record the parabolic traces of the ions. The ion beam was extracted into the TPS by a 100 μm aperture which ensures a pencil-like-beam with negligible transverse velocity and provides a better trace resolution. The TPS is generally kept separately in a differentially pumped vacuum chamber maintained at 2 × 10^−7^ mbar pressure, whereas the main experimental chamber is kept at 4 × 10^−5^ mbar.

## Additional Information

**How to cite this article**: Dalui, M. *et al.* Preferential enhancement of laser-driven carbon ion acceleration from optimized nanostructured surfaces. *Sci. Rep.* **5**, 11930; doi: 10.1038/srep11930 (2015).
